# The emerging role of neutrophils in autoimmune‐associated disorders: effector, predictor, and therapeutic targets

**DOI:** 10.1002/mco2.69

**Published:** 2021-07-22

**Authors:** Xiaohong Fu, Heting Liu, Gang Huang, Shuang‐Shuang Dai

**Affiliations:** ^1^ Department of Biochemistry and Molecular Biology, College of Basic Medical Science Third Military Medical University (Army Medical University) Chongqing China

**Keywords:** autoimmune‐associated disorders, mechanism, neutrophil, targeted therapy

## Abstract

Neutrophils are essential components of the immune system and have vital roles in the pathogenesis of autoimmune disorders. As effector cells, neutrophils promote autoimmune disease by releasing cytokines and chemokines cascades that accompany inflammation, neutrophil extracellular traps (NETs) regulating immune responses through cell–cell interactions. More recent evidence has extended functions of neutrophils. Accumulating evidence implicated neutrophils contribute to tissue damage during a broad range of disorders, involving rheumatoid arthritis (RA), systemic lupus erythematosus (SLE), primary sjögren's syndrome (pSS), multiple sclerosis (MS), crohn's disease (CD), and gout. A variety of studies have reported on the functional role of neutrophils as therapeutic targets in autoimmune diseases. However, challenges and controversies in the field remain. Enhancing our understanding of neutrophils’ role in autoimmune disorders may further advance the development of new therapeutic approaches.

## Graphical abstract

Various roles of neutrophils occur in autoimmune‐associated disorders, including rheumatoid arthritis (RA), systemic lupus erythematosus (SLE), primary sjögren's syndrome (pSS), multiple sclerosis (MS), and crohn's disease (CD). Neutrophils play a bidirectional regulatory role in the autoimmune‐associated disorders and may serve as a predictor of disease progression. Further, increased knowledge of neutrophils’ features can lead to the development of novel therapeutics.

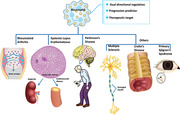

**Abbreviations**
AIA: adjuvant‐induced arthritis
CCR2: CC‐chemokine receptor
CD147: extracellular matrix metalloproteinase inducer
CIA: collagen‐induced arthritis
CXCL1/2: CXC‐chemokine ligand 1/2
CXCL5: CXC‐chemokine ligand 5
CXCL8: CXC‐chemokine ligand 8
Gal‐9: galectin‐9
GITR: glucocorticoid‐induced tumor necrosis factor receptor‐related protein
IL‐17: interleukin‐17
IL‐6: interleukin‐6
JAK: Janus kinase (JAK)‐signal transducer and activator of transcription
Mice (mBSA): mice with methylated bovine serum albumin
MPO: myeloperoxidase
Ncf1**mice: Ncf1 (m1J) mutated mice
NCF1‐339: a single‐nucleotide polymorphism in the NCF1 gene
NE: neutrophil elastase
NET: neutrophil extracellular trap
NOX2: NADPH oxidase 2
PAD4: peptidyl arginine deiminase 4
RIPK1: receptor‐interacting protein kinase 1
ROS: reactive oxygen species
SKG mice: a monogenic model of autoimmune arthritis due to altered signal transduction in T cells

## 1 INTRODUCTION

Autoimmune diseases are a group of disorders characterized by a failure in self‐tolerance to a wide variety of autoantigens.^1^, ^2^ From a clinical perspective, autoimmune inflammation may involve many or even all body organ systems, such as rheumatoid arthritis, systemic lupus erythematosus (SLE) and others (primary sjögren's syndrome [pSS], multiple sclerosis [MS], crohn's disease [CD], and gout).^3^ The pathogenesis of autoimmunity involves dysfunction of the entire immune system, including neutrophils of innate immune, B and T cells of the adaptive immune, dendritic cells (DCs), and macrophages. Within the various cell types related to autoimmune diseases’ pathogenesis, neutrophils are considered to be essential participants in the occurrence and progression of these diseases.^4^

Neutrophils are the earliest cells gathered at infection or inflammation places and account for around 60% of all leukocytes in the circulation.^5^ Recent reports confirm that the average circulating lifespan of mouse‐derived neutrophils extend as long as 12.5 h, while human neutrophils have reached an unprecedented 5.4 days.^6^ The rapid activation of neutrophils and their exponential increase in their lifespan ensure long‐term existence in the inflammation site. In the inflammation process of autoimmune diseases, neutrophils “shoot at first sight” and quickly eradicate invading pathogen infections, thereby playing a host defense role and killing pathogens through various strategies. These strategies include phagocytosis,^7^, ^8^ degranulation, reactive oxygen species (ROS) production, and NETosis process. Degranulation refers to the release of granular antimicrobial peptides by neutrophils, such as myeloperoxidase (MPO),^9^ neutrophil elastase (NE), and matrix metalloproteinases (MMPs).^10^, ^11^ In the case of external stimuli, neutrophils quickly produce ROS and promote oxidative burst.^12^ Through the NETosis process, neutrophils squeeze out the chromatin network structure and bind to the granular peptides of neutrophil extracellular traps (NETs).^4^, ^13^, ^14^ During inflammation, receptors on neutrophils and vascular endothelial cells regulate the adhesion of neutrophils through complex interaction and control the subsequent migration process from the circulation into tissues.^15^, ^16^, ^17^ Data from experimental and clinical settings indicate that the function of neutrophils is not only limited to their roles in acute infection. These breakthroughs will change our understanding of the presence or absence of neutrophils.^18^

Under the general context of inflammation, neutrophils have been extensively and deeply studied. Ample evidence indicates that neutrophil promotes inflammation and contributes to the pathogenesis of autoimmune diseases.^19^, ^20^, ^21^, ^22^ In this study, we have reviewed a large number of relevant literatures and summarized the latest recent advances in neutrophils, focusing on their roles and functions in distinct autoimmune disorders, and their targeted therapies for neutrophil‐derived disorders. Furthermore, we hope to draw attention to the modulatory role of neutrophils in autoimmune‐associated disorders, which will help us understand the role of neutrophils in autoimmune disorders, and elucidate the etiology, prevention, and treatment of autoimmune disorders.

## 2 NEUTROPHILS IN RHEUMATOID ARTHRITIS

### 2.1 Role and function

RA is a chronic autoimmune arthropathy mainly characterized by joint damage caused by an inflammatory reaction, which leads to joint dysfunction and disability and seriously affects patient life quality.^23^ Neutrophils are abundant in both synovial tissue and fluid, supporting an essential role for these cells in the initiation and progression of RA.^24^, ^25^ The pathogenesis of neutrophils in RA relates to changes in multiple stages, like regulating the functions of other immune cells; acts as APCs by directly interacting with T and B cells; cell–cell interactions; and accelerate the release of proteases and NETs.^26^

In inflammatory conditions, neutrophil migration may be induced by cytokines through further secreting other cytokines and chemokines. CC‐chemokine receptor (CCR2),^27^ CXC‐chemokine ligand 1 (CXCL1),^28^ CXC‐chemokine ligand 5 (CXCL5),^29^, ^30^ CXC‐chemokine ligand 8 (CXCL8),^31^ CCL3,^32^ TNFα,^33^ and IL‐1β^34^ are several essential cytokines/chemokines that are recruited by neutrophils. Talbot et al. found that CCR2 has directly participated in the detrimental infiltration of neutrophils into the joints in RA patients.^35^ It has been well documented that CXCL1 can induce proinflammatory gene expression in synergy with interlukin (IL)‐17A and IL‐17F.^36^, ^37^ Besides, CXCL5 reported being as crucial as CXCL8, similar to neutrophil chemoattractant in RA synovial fluid, with immuno‐neutralization eliminating over 40% of chemotaxis. IL‐33 could stimulate activated synovial cells and macrophages to generate cytokines such as TNFα, IL‐1β, CXCL, and CCL3, which will recruit neutrophils to the joint in a cascade manner. Alternatively, IL‐33 directly draws neutrophils to the places of inflammation. Both monocyte chemoattractant protein 1 (MCP‐1) and IL‐8 work for the recruitment of inflammatory cells into the arthritic joint.^38^ These examples indicate the significance of cytokines/chemokines for recruiting neutrophils into the joints during the acute phase of early RA. In response, these recruited neutrophils polarize and crawl along with the endothelial cells and quickly migrate into inflamed tissues, thereby identifying and engulfing pathogens, such as bacterias, viruses, and fungi and acting as a guardian to defend lifeline.

Notably, the inflammatory microenvironment is the stimulus that allows neutrophils to gain APC‐like capabilities. A large number of findings powerfully demonstrate that neutrophils also serve as APCs by directly interacting with T and B cells, further promoting T‐cell activation, migration, and DC maturation. Furthermore, T cells promote the differentiation of neutrophils into APCs.^39^ In the form of APCs, neutrophils present antigens and activate adaptive immunity. Then, it enhances the expression of plasma membrane receptors (MHC class II antigens) to push more neutrophils to present antigens to T cells.

One of the main characteristics of neutrophils’ defensive function is its ability to form NETs. NETs’ formation involves activating neutrophils to release their chromatin (DNA and its related histones), carry granzymes and extracellular network structure that can capture and kill extracellular organisms.^40^ Recently it has been reported that the release of NETs represents a new form of neutrophil death (termed NETosis) that is different from apoptosis and necrosis and provides a way for neutrophils at the end of their lifespan to continue to kill pathogens. Interestingly, recent studies have found that neutrophils need autophagy to maintain their normality and fulfill its efficacy,^41^ which has been confirmed by enhancing of the autophagy ability in RA patients.^42^

Neutrophils are the most abundant cell type and detected in RA synovial fluid and synovial tissue. The complex and diverse molecule mechanisms caused by neutrophils have largely contributed to the continuous inflammation and tissue damage of RA synovial joints, and further support the important role of neutrophils in driving the pathogenesis and manifestations of RA.

### 2.2 Neutrophil‐directed therapy in RA

Currently, wide‐ranging used targeted neutrophil therapeutics mainly neutralize specially related cytokines that drive the occurrence of RA. Among them, many cytokines directly affect the function of neutrophils. The effect of targeted therapy depends on the degree of reduction of neutrophil‐mediated inflammation. Infliximab (INF), methotrexate (MTX), leflunomide, and glucocorticoids are several common nonbiologic disease‐modifying antirheumatic drugs, which are mainly effective by changing neutrophil functionality through various mechanisms.^43^ For example, INF^44^ suppresses neutrophil chemotaxis and the production of ROS by blocking the priming activation of monocytes on neutrophils. Nowadays, MTX has become a common prescription drug for rheumatoid arthritis. Compared with nonresponders, treatment of RA with MTX alone can reduce the production of neutrophil ROS and supplement system activity in patients with MTX‐responder, showing that disease activity (DAS‐28) and inflammatory mediators (CRP) decreased. Both i‐RA and a‐RA patients present functional alterations in neutrophils and complemented system.^45^ And interestingly, INF and MTX combination therapy reduced the Ca^2+^ concentration significantly by blocking the TRPM2 channel, which makes INF and MTX useful antagonists of neutrophil apoptosis and mitochondrial oxidative stress in RA patients. Also, targeting the cytokines TNF and IL‐6 reduced the production of ROS and changed the migration characteristics of neutrophils in RA.^46^

Various experimental treatments mainly target diverse cytokines and their receptors, form extracellular trap of neutrophils, and use neutrophil microparticles to influence the function of neutrophils. During phase 3 and 4 clinical trials, treatment with tocilizumab (TCZ, an inhibitor of IL‐6 receptor‐α) can reduce the number of neutrophils and the incidence of infection in RA patients.^47^ The downregulation of peptidyl arginine deiminases (PADs) mediates the effect of iguratimod and inhibits the expression of CPs in neutrophils.^48^ Therefore, strategies to inhibit PADs are promising in mouse models.^49^ Filgotinib, as a selective Janus kinase (JAK)‐1 inhibitor, has shown initial efficacy and encouraging safety profile in randomized phase IIa trials. Neutrophil exosomes are responsible for the main anti‐inflammatory effects in RA, and neutrophil decoy microparticles are being developed into nanotechnology, which can supply local therapy options for certain RA patients. Nanoparticles coated with neutrophil membrane show significant therapeutic effect works by improving joint damage and suppressing the severity of overall arthritis.^50^

## 3 NEUTROPHILS IN SYSTEMIC LUPUS ERYTHEMATOSUS

### 3.1 Role and function

SLE is a chronic autoimmune disorder, which is mainly characterized by the activation of inflammatory immune cells and the production of proinflammatory autoantibodies, leading to self‐attack of organs, tissues, and cells.^51^ Neutrophil dysregulation is related to SLE's pathogenesis, abnormal neutrophils fueling inflammatory states by producing disease‐inducing cytokines (IL‐1β or BLyS) and becoming a source of many autoantigens in the disease through NETosis.^52^

In SLE, NET formation causes autoantigens to leak from the uncleared cell debris.^53^ Then, follicular dendritic cells in the germinal centers of secondary lymphoid tissues present these neo‐epitopes to B cells, thereby destroying self‐tolerance. Activation of autoreactive B cells and subsequent autoantibodies’ production promotes the formation of immune complexes (ICs), which can cause inflammation reaction and lead to further tissue injury. ICs can also absorb phagocytes and subsequently yield more proinflammatory cytokines. These processes create a vicious circle, leading to continuous inflammation. A great number of autoepitopes of NET proteins that are recognized by autoantibodies produced by patients with SLE can also be removed by proteases. In SLE and non‐SLE subjects, the enhancement of NET formation is associated with vascular disease, which may accelerate the construction of coronary plaque and lipoprotein disorders.^54^ NETs can drive macrophages and other cells to release IL‐1 and IL‐18 through NLRP3 inflammasome or P2X7 purinergic receptors, thereby further exacerbating the inflammatory state of SLE patients.^55^ Fc receptor (FcγR1 or CD64) expression on neutrophils is a potential biomarker of bacterial infections.^56^

Neutrophils ingest more secondary necrotic cells (SNECs), which makes phagocytes NADPH oxidase 2 (NOX2 complex) generate low ROS, resulting in the detrimental inflammatory environment and deteriorated disease status.^57^ Meanwhile, when uncleared apoptotic cells lose their membrane integrity, they present autoantigenic molecules in an inflammatory milieu. Dead cells are insufficiently removed, leading to an autoimmune response against components of the cell's nucleus.

Recent findings indicated that the susceptibility of SLE is related to low production of ROS. NCF1‐339 genotype (single‐nucleotide polymorphism of NCF1 gene [rs201802880], encoding NADPH oxidase type II subunit NCF1/p47^phox^) affects the formation of NET, lowering the production of ROS.^58^ ROS production deficiency can lead to excessive IFN response, earlier disease onset, and higher sensitivity to SLE.^59^

Another SLE feature is the elevated level of a pathogenic neutrophil subset, called low‐density granulocytes (LDGs).^60^ SLE LDGs display significant transcriptional and epigenetic heterogeneity and comprise two subpopulations of intermediate‐mature and immature neutrophils, with different chromatin accessibility and differences in transcription factor motif analysis. Differences in NET formation, oxidized mitochondrial DNA release, chemotaxis, phagocytosis, degranulation, ability to harm the endothelium, and IFN stimulation responses are evident among LDG subsets. Neutrophils can produce type I interferon and can also be used as an antigenic trigger to induce IFNα production.^59^

It has also suggested that differential methylation found on specific genes, and the most prominent feature of SLE is the decreased methylation of genes in the IFN system. Genetic variations in the A20 DUB domain provide a genetic link to citrullination and NETs in SLE.

### 3.2 Neutrophil‐directed therapy in SLE

According to the meta‐analysis, compared with healthy controls, the ratios of neutrophil to lymphocyte (NLR) and platelet to lymphocyte (PLR) of SLE patients are significantly higher and are positively correlated with SLE disease activity index (SLEDAI), suggesting that NLR and PLR can be used as potential biomarkers in SLE management.^61^ Besides, NETs are the source of extracellular HMGB1. The expression of HMGB1 in NETs is higher among patients with lupus nephropathy (LN), which is associated with clinical and histopathological characters of active nephritis, suggesting that this alert protein may play a role in the pathophysiology of kidney damage in SLE.^62^

PAD4 (peptidyl arginine deiminase‐4), one of the enzymes that mediate the citrullination of arginine, plays an essential role in the formation of NET after receiving specific type of stimulation. Although the part of PAD4 in the clinical and immunological behavior of mouse SLE is debated,^63^, ^64^ inhibition of PAD4 would have special treatment effects in humans. Therefore, PAD4 may become a potential central drug target through which it could improve NET‐induced atherosclerosis.

Light of the ultraviolet‐visible (UV‐VIS) spectrum will trigger and aggravate the severity of SLE. Avoiding light exposure is a treatment to alleviate the condition because the light‐induced NETosis process relies on the production of extracellular ROS caused by riboflavin excitation and its subsequent reaction with tryptophan. Light‐induced NETosis required activation of NE and MPO and induced histone citrullination.^65^

L‐ascorbic acid‐a (HIF‐1α inhibitor) or bosentan (an endothelin‐1 receptor antagonist) used to lessen the release of NET targeted treatment for neutrophils, revealing that the inhibition of endothelin‐1 and HIF‐1α could significantly suppress the “pre‐NETotic” step of NET formation in human SLE.^64^ Hydroxychloroquine, a disease‐modifying antirheumatic drug and a late‐stage autophagy inhibitor, is used to prevent plaque formation and increase the survival rate of SLE patients. The NET formation could be targeted by autophagy inhibition with hydroxychloroquine.^66^ Similarly, nocodazole targeting neutrophils in SLE patients could reduce the release of chromatin by interfering with the polymerization of tubulin into microtubules or cytochalasin D (an inhibitor of actin filamentation). M1/70 (an integrin adhesion receptor antibody) pretreatment of neutrophils would reduce the distribution of chromatin to NETs.^67^

## 4 OTHERS

In addition to these common disease categories, autoimmune diseases also include some small crowd diseases, including pSS, MS, CD, and gout, etc.^68^ As an essential part of the immune system, neutrophils are dedicated to mobilizing immune defense. Neutrophil dysregulation is a shared mechanism that plays a crucial role in the pathogenesis of these diseases.

### 4.1 Neutrophils in pSS

Neutrophils mainly contribute to ROS products during the respiratory burst, which may increase the degree of inflammatory and fibrotic processes, and they participate in the endothelial damage of pSS.^69^ Compared with controls, pSS patients have increased basal neutrophil adhesion, which suggests that blood neutrophils are activated.^70^ However, in those with the recurrent bacterial infection, the adherence of neutrophils in patients significantly decreases, and the opsonic activity of plasma also reduces. In contrast, the contents of neutrophils in intracellular lactoferrin and lysozyme were normal. Correlation analysis found that elevated level of neutrophils and hypoproteinemia in the pSS cohort are significantly associated with lung infections (*p* < 0.05).^71^ Neutrophils were increased considerably in the salivary glands of pSS patients and correlated with focus score.^72^ Neutrophil/lymphocyte ratio (NLR) as an inflammation index and red blood cell distribution width (RDW) may prove useful indices to estimate pSS disease activity.^73^ Neutropenia should be added to various hematological manifestations of pSS, and it may be a presenting feature and an important clue to the diagnosis.

Owing to the complexity of pathogenesis and heterogeneity behind the clinical manifestations, many targeted immunomodulatory therapies for pSS have not shown any benefits in clinical trials. To date, no specific treatment for this disease has been approved. We have made little progress in targeted immunotherapy for pSS, and most of the time, it is still based on empirical therapy. For those patients with pSS, conventional DMARDs or biologics (mostly rituximab) are still mainstream treatment.^74^, ^75^ Clinically, reasonable use of epratuzumab and rituximab based on the patient's condition has shown a certain therapeutic effect. Ianalumab (VAY736), as another medicinal drug for pSS, eliminates pathogenic B cells through directly lysing B cells and breaking BlyS and its receptor signaling pathway, which has remarkably improved the clinical parameters and laboratory index of patients.

### 4.2 Neutrophils in MS

Mounting evidence shows the role of neutrophils in MS. For example, neutrophils in the cerebrospinal fluid increased in pediatric MS patients, and MPO (neutrophil product) elevated in serum in MS.^76^ Resting circulating neutrophils begin to sensitize after encountering pathogenic stimuli, which triggers the upregulation of surface receptors and induces chemokine receptor in MS (G‐CSF, CXCL1, CXCL8, CXCL5, fMLPR14, TLR215, NE) to increase significantly. Studies have found that neutrophils are related to the level of the cytokine IL‐17A (supporting neutrophil activation and recruitment) during the early relapse of MS patients.^77^ Furthermore, neutrophils specifically express the aspartic retroviral‐like protease ASPRV1, which increased in the central nervous system during severe cases of MS.^78^ These observations could collectively support that neutrophil activity is a factor that influences MS development and severity.

At present, there are no direct target neutrophil drugs for the treatment of MS, and a very small number can play a therapeutic role by affecting neutrophils. Fingolimod (sphingosine analog) is an immunomodulatory drug that causes a decline in the neutrophil count after consistent dosing.^79^ The immune‐suppressive dimethyl fumarate may act to alter neutrophil function.^80^ Cladribine, cyclophosphamide, and mitoxantrone, as cell cycle inhibitors, disrupt DNA synthesis of lymphocytes and other cells, and each reduces the number of neutrophils.^78^, ^81^, ^82^ The deficiency of therapeutic drugs emphasizes further study of the role of neutrophils in MS.

### 4.3 Neutrophils in Crohn's disease

CD is a chronic inflammatory disorder that mainly affects gastrointestinal tract.^83^ In the early stages of CD, a massive influx of neutrophils swallows and kills invading bacteria and fungi and digests foreign organic matters. The innate immune response delays the recruitment of neutrophils to the body's trauma, which has been confirmed in later stages of CD patients. Discovered by whole‐exome sequence analysis, neutrophils produce low levels of ROS, and specific changes occurred in genes that regulate glucose metabolism and antimicrobial reactions.^84^ This may be related to the impaired recruitment of neutrophils and killing of bacteria by luminal neutrophils, causing mice to increase their susceptibility to CD disease.^85^

Studies have shown that CD's defect is related to the entry of neutrophils into damaged tissues rather than into the circulation. Therefore, using granulocyte–macrophage colony‐stimulating factor (GM‐CSF) to increase the number of circulating neutrophils to treat CD is invalid.^86^, ^87^ Recent evidence shows that IgA has anti‐inflammatory properties that can be therapeutically exploited. IgA can induce neutrophil death, which may involve at least in part FcaRI (CD89) and Mac‐1 (CD3 and CD11b/CD18) signaling.^88^

### 4.4 Neutrophils in gout

Although gout has not been well demonstrated as an autoimmune disease, it has similar clinical symptoms and pathogenesis to some autoimmune diseases. Patients with RA, SLE, and gout suffer from hyperuricemia and reduced amino acid content, which is the purine's precursor. The close correlation between these patients and the purine index has been observed.^89^ Neutrophil granulocytes contain many proinflammatory substances and mechanisms that enable them to drive local acute immune responses to invade microorganisms or become endogenous inflammatory triggers.^90^ Neutrophil mediated the production of interleukin‐1β (IL‐1β), which does gouty attacks, causing joint destruction, intense pain, and fever.^91^ The regulation of inflammation derived by inflammasome mediated by neutrophil‐microvesicles (PMN‐Ecto) is a puzzling concept: in the early periods of inflammation, neutrophils infiltrate the peritoneum in response to phosphatidylserine (PS)‐positive PMN‐Ecto released by C5a.^92^ The precipitation of uric acid causes the inflammation in gout, these crystals mediate the activation of inflammasomes in local immune cells and further lead to the rapid recruitment of neutrophils. During an acute gout attack, this massive influx of neutrophils is accompanied by notoriously clinical inflammatory symptoms.^93^ Activated neutrophils form NETs, which is found abundantly in gout patients’ synovial fluid, and this process is associated with autophagy and IL‐1β. Studies have shown that a known alternative metabolic fuel β‐hydroxybutyrate (BHB) is also an anti‐inflammatory molecule and can be used as a treatment for gout.^90^ The main mechanism is that BHB suppresses the NLRP3 inflammasome in neutrophils through reducing the initiation and assembly steps. In turn, it blocks the generation of IL‐1β in mouse and human neutrophils. In most gouty patients, functional activity of neutrophils is impaired due to their lower phagocytic function, which inhibits the body's antioxidant defense capabilities and leads to a chronic sustained pattern of the inflammatory process.

## 5 DISCUSSION

**FIGURE 1:**
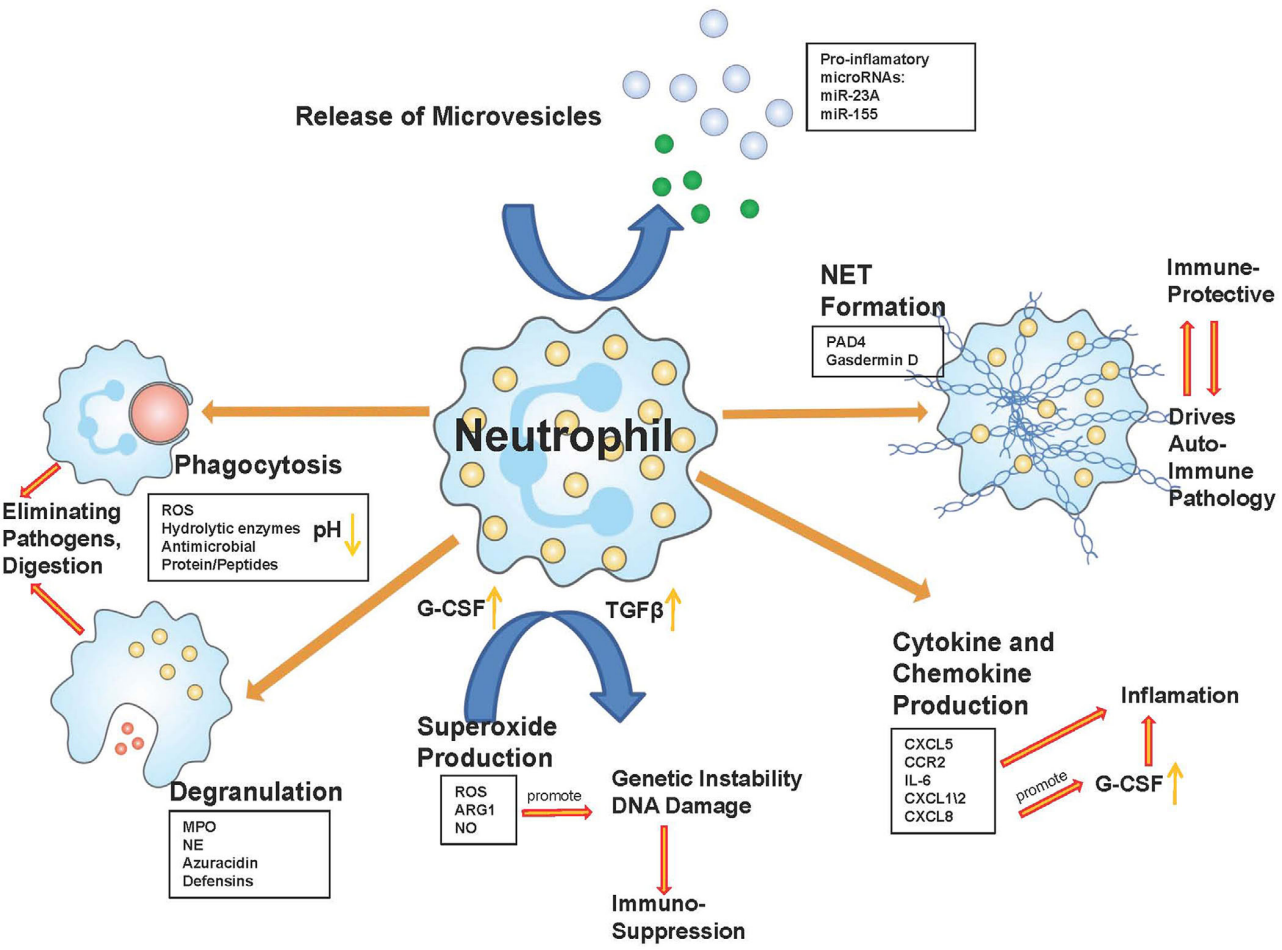
The role of neutrophils in autoimmune disorders. Neutrophils are involved in a variety of immune and inflammatory processes, where they can eliminate the invading pathogens through endocytosis and degranulation. They can also drive the immune process or immune suppression by generating reactive oxygen species (ROS), recruiting cytokine and chemokine, and neutrophil extracellular trap (NET) formation

Autoimmune diseases involve complex immunity disorders that cause loss of self‐tolerance and attack endogenous tissues and cells. Neutrophils acting as instrumental immune cells in the autoimmune disease, infiltrating tissue through immune complex and complement‐mediated mechanisms, participating in the release of proteolytic enzymes, and causing tissue damage inflammation.^3^, ^4^, ^94^ However, even though neutrophils are known to be at the center of disease pathogenesis, the mechanism behind this is not fully understood, and targeted drugs have not been studied in detail. In this study, we tried to expand our understanding of the relationship between neutrophils and autoimmune diseases through comprehensive literature reviews. We also reviewed the core functions of neutrophils between innate and adaptive immunity (Figure 1).

Neutrophils participate in the initiation of the inflammatory response through a variety of mechanisms.^91^, ^92^ These mechanisms highlight that neutrophils participate in the pathogenesis of disease in multiple ways, not just by releasing proteases and ROS to induce tissue damage. Ample evidence indicated that neutrophil‐derived mechanisms not only exacerbate autoimmune diseases but also include cases where neutrophils protect the host from inflammatory tissue damage. Neutrophils exert regulatory function in the overall immune response by direct production of cytokines/chemoattractants and indirect effect between other immune cells (such as MPO and ROS agents) (Table 1). It is also clear that neutrophils are the source of self‐antigens in many autoimmune diseases. Studies on a large number of animal models have explained most of this complexity. Improvement of neutrophil‐specific gene targeting and interventional approaches will further reveal the complexity. In the past, drug development work always avoided targeting neutrophils, mainly due to concerns about severe infectious complications. As we now know, because of the functional role of neutrophils in the pathogenesis of autoimmune disorders, it is possible to envision the development of drugs that can block the function of neutrophils while only moderately impairing the host's defenses. Using existing animal models and new ones development to alter the position of neutrophils, which is essential to improve their ability to control autoimmune diseases.

**TABLE 1:** Effector molecules of neutrophils in autoimmune diseases

Diseases	Effector molecules	Functions	Species	References
RA	CCR2	Infiltration of neutrophils into the joints	Mice with adjuvant‐induced arthritis (AIA)	^27^, ^35^
	CXCL5	Citrullinated ENA‐78/CXCL5 recruiting monocytes to inflamed joint tissues	Humans and mice with AIA	^30^
	GITR	Glucocorticoid‐induced tumor necrosis factor receptor‐related protein (GITR) triggering is required for the development of immune response against CII	Collagen‐induced arthritis (CIA)	^95^
	miR‐451	Suppresses neutrophil chemotaxis	Humans and SKG mice	^96^
	IL‐17	Induces hypernociception and neutrophil migration	Mice (mBSA)	^97^
	IL‐17B	Controls immune cell trafficking and neutrophil homeostasis in the inflamed tissues	Humans	^36^
	CD147	Upregulates calcium‐induced chemotaxis, adhesion ability, and invasiveness of human neutrophils	Humans	^98^
SLE	NCF1‐339	Alters formation of neutrophil extracellular traps, high serum interferon activity, and antiphospholipid syndrome	Humans	^99^
	NCF1‐339	Reduces oxidative burst	Humans	^58^
	NOX2 complex	Produces low ROS	Ncf1**mice	^100^
	TANK‐binding kinase1 (TBK1)	Activates IRF3 and IRF7, leading to IFN‐I production and subsequent induction of interferon‐stimulated genes (ISGs)	Humans	^101^
	RIPK1	Decreasing neutrophil death and formation of NETs	Humans	^102^
	Gal‐9	Inhibits TLR7‐mediated autoimmunity	Spontaneous murine models of lupus (i.e., BXSB/MpJ and NZB/WF1 mice)	^103^

**FIGURE 2:**
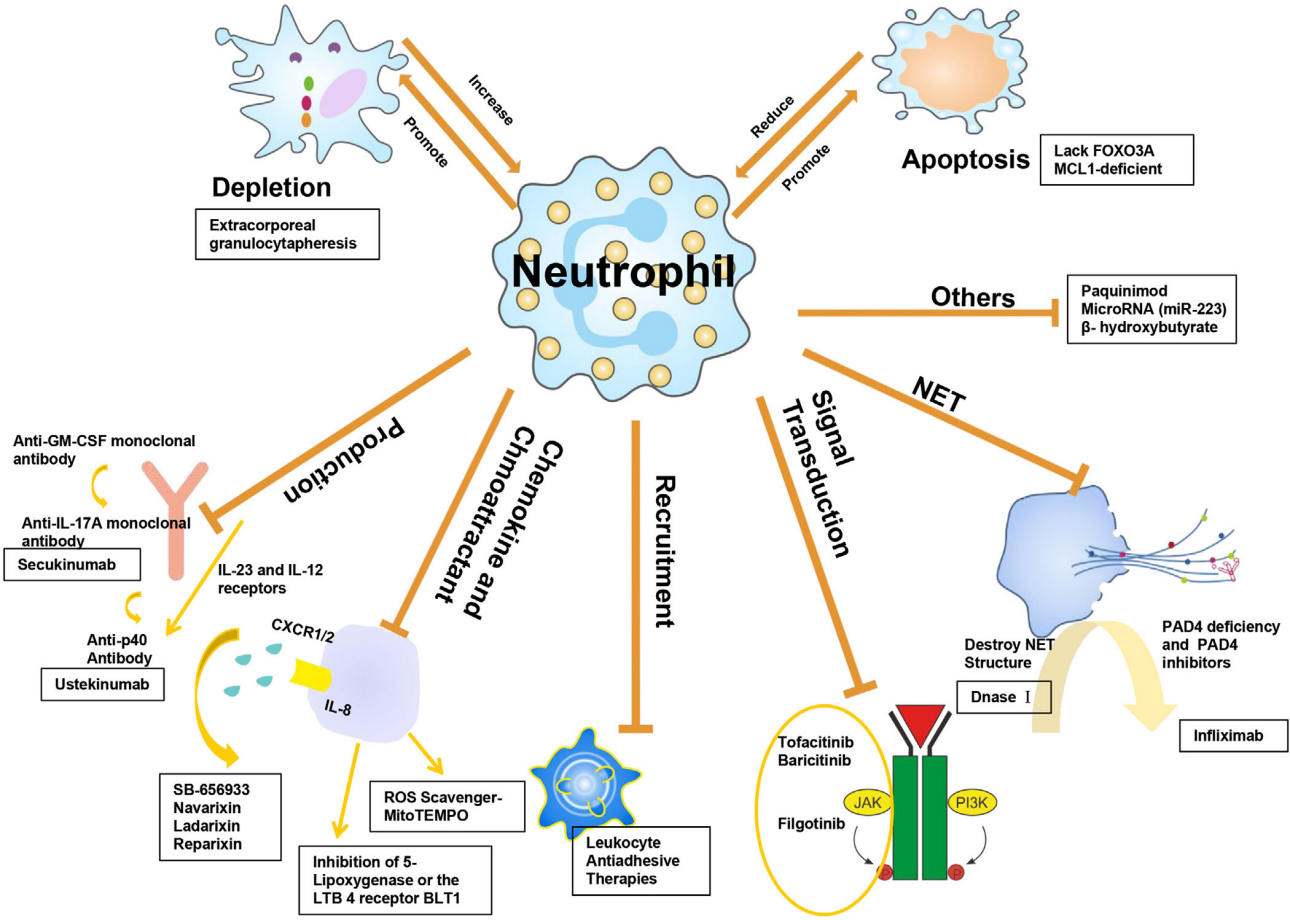
The therapeutic strategies of neutrophils in autoimmune disorders. Several aspects of neutrophil biology may be therapeutically targeted, including promotion of depletion and apoptosis, and targeted inhibition of neutrophil production, recruitment of chemokines, signal transduction pathways, and neutrophil extracellular trap (NET) formation, etc. In some cases, increasing depletion or reducing apoptosis is the aim. The boxes indicate representative drugs/strategies

Our current understanding of neutrophil characteristics is limited, and even the detection of marker effects (such as NETosis) is still in its infancy. Nevertheless, with the rapid development of multidimensional and single‐cell analysis techniques, it is only a matter of time before this technical problem is solved.^104^ Currently, it is being actively studied whether neutrophils, their subgroups, or specific effector functions can be used as biomarkers. The manipulation of neutrophils may help treat autoimmune diseases in the future and even become the basis for new targeted drugs (Figure 2).

Recognizing neutrophils’ regulatory roles in autoimmune diseases will encourage us to reconsider the biological significance of neutrophils in physiological and pathological conditions. In addition, based on neutrophils’ ability to regulate autoimmune diseases and the development of more and effective targeted drugs for autoimmune diseases, it may potentially provide new therapies for autoimmune disorders, such as RA, SLE, and other diseases.

## CONFLICT OF INTEREST

The authors declare that there is no conflict of interest.

## ETHICS STATEMENT

Not applicable.

## AUTHOR CONTRIBUTIONS

Xiaohong Fu, Gang Huang, and Shuang‐shuang Dai designed the study and wrote the manuscript. Heting Liu contributed to the conception of the study and correction of the manuscript.

## Data Availability

No new data were created or analyzed in this review. Data sharing is not applicable to this article.
